# Tensile Properties and Microstructure Evolutions of Low-Density Duplex Fe–12Mn–7Al–0.2C–0.6Si Steel

**DOI:** 10.3390/ma15072498

**Published:** 2022-03-28

**Authors:** Shuai Liu, Yinlei Ge, Huanyou Liu, Junyu Liu, Yunli Feng, Chen Chen, Fucheng Zhang

**Affiliations:** 1 College of Metallurgy and Energy, North China University of Science and Technology, Tangshan 063210, China; geyinlei1988@163.com (Y.G.); huanyouliuncst@163.com (H.L.); junyuliuncst@163.com (J.L.); yunlifeng1989@163.com (Y.F.); fuchengzhang1124@163.com (F.Z.); 2 State Key Laboratory of Metastable Materials Science and Technology, Yanshan University, Qinhuangdao 066004, China; cchen_ysu@163.com

**Keywords:** Fe–Mn–Al–C steel, dualphase steel, lowdensity steel, δferrite, stacking fault energy

## Abstract

An austenite-ferrite duplex low-density steel (Fe–12Mn–7Al–0.2C–0.6Si, wt%) was designed and fabricated by cold rolling and annealing at different temperatures. The tensile properties, microstructure evolution, deformation mechanism and stacking fault energy (SFE) of the steel were systemically investigated at ambient temperature. Results show two phases of fine equiaxed austenite and coarse band-like δ-ferrite in the microstructure of the steel. With increasing annealing temperature, the yield and tensile strengths decrease while the total elongation increases. At initial strains, the deformation is mainly concentrated in the fine austenite and grain boundaries of the coarse δ-ferrite, and the interior of the coarse δ-ferrite gradually deforms with further increase in the strain to 0.3. No twinning-induced plasticity (TWIP) or transformation-induced plasticity (TRIP) occurred during the tensile deformation. Considering element segregation and two-phase proportion, the chemical composition of austenite was measured more precisely. The SFE of the austenite is 39.7 mJ/m^2^, and the critical stress required to produce deformation twins is significantly higher than the maximum flow stress of the steel.

## 1. Introduction

Automotive lightweight design is the main development direction of the automotive industry. Lightweight design can be achieved by two main methods: (1) improving the strength of automotive steel and reducing steel consumption whilst ensuring automotive safety [1,2,3] and (2) using lightweight materials, such as aluminum, magnesium alloys, plastics or carbon fiber composites. However, lightweight materials often have disadvantages, such as high cost, low strength and poor thermal stability, which largely limit their application in the automotive industry [4]. In recent years, researchers have focused on a new strategy, that is, the development of low-density, high-strength and high-plasticity steel. A certain amount of light Al element is added to the steel to develop new Fe–Mn–Al–C grade low-density steels. Fe–Mn–Al–C steels possess high strength and plasticity (400 MPa ≤ yield strength ≤ 1000 MPa, 600 MPa ≤ ultimate tensile strength ≤ 2000 MPa and 30% ≤ total elongation ≤ 100% [5,6,7,8]), good fatigue resistance [9,10] and high toughness [11,12]. This new type of automotive steel has great potential and has become increasingly popular in recent years.

The primary purpose of the addition of Al is to reduce the steel density. Frommeyer et al. reported a linear relationship between Al content and steel density, that is, a 1.5% reduction in density per Al addition of 1 wt% [13]. In addition to weight reduction, Al plays a role in increasing the stacking fault energy (SFE), and the deformation mechanism of the Fe–Mn–Al–C steel changes with increasing Al content [14,15]. Fe–Mn–Al–C steel has a ferrite, austenite or even multiphase structure depending on the content of Al [16]. Al is a strong ferrite-stabilized element. When a large amount of Al is added to the Fe–Mn–Al–C alloy system, δ-ferrite formed by liquid–solid phase transformation at high temperatures does not completely disappear during subsequent solid-phase transformation [17,18]. Due to the presence of δ-ferrite, a mixture of δ-ferrite and martensite replacing the full martensite in conventional welds improves the welding performance of steel [19]. However, δ-ferrite is shaped as a coarse dendrite in the as-cast microstructure and becomes coarse band-shaped in the subsequent hot rolling process. Coarse δ-ferrite has an adverse effect on the properties of steel. Zhang et al. [20] studied the effect of solution treatment on the mechanical properties of Fe–18.1Mn–9.6Al–0.65C steel. They found that band-shaped δ-ferrite grains can be broken, separated and distributed uniformly on the austenite matrix by proper solution treatment, thereby improving the microstructure uniformity and mechanical properties. In Fe–Mn–Al–C steel, the contents of C and Al play an essential role in the formation of κ-carbide [21,22]. When κ-carbide is precipitated in the grains, the yield and tensile strength are improved by precipitation strengthening. When the Al content is higher than 5.5% and the C content is higher than 0.7%, the ductility and toughness of the steel will be reduced due to the precipitation of intergranular κ-carbide.

In this study, a low-carbon duplex δ-ferrite and austenite low-density steel (Fe–12Mn–7Al–0.2C–0.6Si, wt%) was designed. Reducing the content of C in the alloy could reduce the possibility of the formation of coarse intergranular κ-carbides and enhance weldability. The addition of Si (0.6 wt%) played a role in solution strengthening and inhibited carbide precipitation. The as-cast or hot-rolled Fe–Mn–Al–C steel usually contained coarse δ-ferrite, which deteriorated the mechanical properties of the steel. Cold rolling and annealing at different temperatures were employed to refine the microstructure and further improve the mechanical properties of the steel. The tensile properties, microstructural evolution, deformation mechanism and SFE of the steel were systematically investigated.

## 2. Materials and Methods

Experimental steel was prepared in a vacuum induction melting furnace. The ingot was homogenized at 1200 °C for 4 h and hot-forged into a square billet with a cross-section of 35 × 45 mm. The chemical composition of the hot-forged billet was measured to be as follows: Mn, 12.8 wt%; Al, 6.8 wt%; Si, 0.63 wt%; C, 0.22 wt%; and Fe, balance. The forged billet was cut into plates of 4 mm thickness and then cold rolled to 2 mm with a reduction of 50%. The cold-rolled steel plates were annealed at 700–1000 °C for 30 min and quenched in water to prevent the precipitation of carbide. Samples with a gauge size of 14 × 5 × 2 mm were cut from the annealed plates along the rolling direction (RD). Room-temperature tensile properties were tested using an Instron-3382 test machine (Instron, a Division of lllinois Tool Works Inc, Norwood, MA, USA) with the nominal strain rate of 2 × 10^−3^ s^−1^. The tensile properties of all samples were determined as follows: the yield strength (YS) is the stress at which a 0.2% plastic deformation is produced, the ultimate tensile strength (UTS) is the maximum flow stress of the steel and the total elongation (TE) is measured by mechanical extensometer. The sample size and tensile test method followed the China National Standard GB/T 228-87. Additional tensile tests were performed and interrupted at strains of 0.15 and 0.3 to examine the microstructural evolutions.

An optical microscope (OM, Zeiss Axiovert 200, Carl Zeiss AG, Oberkochen, Germany) and electron backscatter diffractometer (EBSD, EDAX-TSL, AMETEK, Inc, Berwyn, PA, USA) were employed to observe the microstructures. Samples for the OM were cut from the annealed plates, mechanically ground, polished and etched by a mixed solution of 4% nitric acid alcohol. EBSD observations were carried out on the RD–normal direction (ND) section of the tensile samples. Samples for the EBSD were cut from the parallel section of the tensile samples and prepared by standard metallographic procedures. The samples were further mechanically polished with SiO_2_ solution, and a remarkably longer polishing time was required to effectively remove the stress layer on the surface. An acceleration voltage of 30 kV, a working distance of 15 mm and a step size of 100 nm were applied during the EBSD observations. The distribution of elements in the different phases of the steel was analyzed by a scanning electron microscope (SEM, FEI Quanta-650 FEG, FEI Company, Hillsboro, OR, USA) equipped with an energy-dispersive spectrometer (EDS, EDAX-GENESIS, AMETEK, Inc, Berwyn, PA, USA). For the SEM and EDS observations, the samples were cut from the annealed plates, prepared via standard metallographic procedures and etched with 4% nitric acid alcohol. An acceleration voltage of 15 kV and a working distance of 10 mm were utilized. An X-ray diffractometer (D/max-2500/PC, CuK_α_) was used to analyze the phase of the steel. X-ray diffraction (XRD, Rigaku Ultima IV, Akishima, Tokyo, Japan) measurements were conducted between 40 and 100° at room temperature using a diffractometer with a Cu target and a scanning rate of 1.5°/min.

## 3. Results

### 3.1. Annealed Microstructure

**Figure 1:**
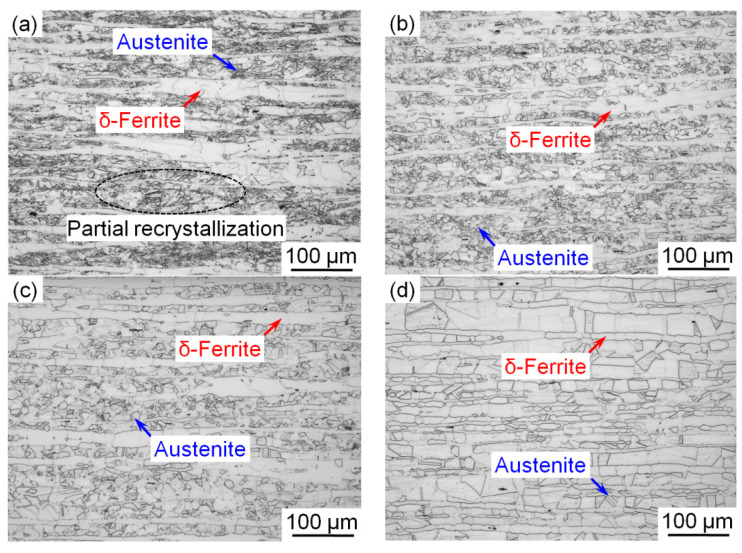
Optical microstructure of the cold-rolled steel annealed at (**a**) 700 °C, (**b**) 800 °C, (**c**) 900 °C and (**d**) 1000 °C for 30 min. Red and blue arrows indicate δ-ferrite and austenite.

Figure 1 shows the metallographic structure of the steel after cold rolling and annealing at different temperatures (700–1000 °C). When the cold-rolled steel was annealed at a lower temperature of 700 °C, a partially recrystallized microstructure, consisting of un-recrystallized and recrystallized austenitic grains, was observed as shown in Figure 1a. By contrast, fully recrystallized austenitic microstructures were obtained when the steel was annealed at temperatures above 800 °C (Figure 1b–d). Two typical microstructures of equiaxed austenite and band-like δ-ferrite were detected in the recrystallized structure of the steel. The average recrystallized grain size of the austenite was measured in the middle of the sample. Five optical micrographs were examined, and approximately 450 measurements were made for each sample. The average grain size of the austenite increased from ~6.6 to ~60 μm when the annealing temperature increased from 800 to 1000 °C. By contrast, the size of the band-like δ-ferrite barely changed with increasing annealing temperature.

**Figure 2:**
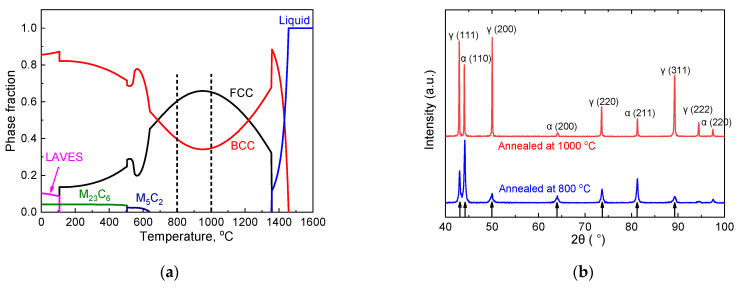
(**a**) Phase fractions of the steel based on an equilibrium thermodynamic analysis as predicted by Thermo-Calc and (**b**) the XRD pattern of the steel annealed at 800 and 1000 °C.

Thermo-Calc thermal simulation software was used to analyze the phase evolution of the steel during the equilibrium cooling process. Figure 2a indicates that the austenite content (FCC) could not reach 100% at any temperature. Thus, the high-temperature δ-ferrite (BCC) phase was not completely transformed into austenite during the cooling process. The high Al content in the experimental steel, which increased the ferrite stability, preserved the high-temperature δ-ferrite to room temperature leading to duplex δ-ferrite and austenite microstructure in the experimental steel [17]. In addition, when the steel was cooled to below 1000 °C, the austenite content in the steel decreased with the temperature. According to Thermo-Calc calculation, the volume fraction of the austenite in the steel at 800 and 1000 °C reached 59.6% and 65.3%, respectively, as shown by two dotted lines in Figure 2a.

The XRD phases of the steel annealed at 800 and 1000 °C were analyzed (Figure 2b). Ferrite and austenite phases were found in the steel. Given that the steel was directly quenched into the water after annealing, no significant carbide precipitation was observed. According to the XRD patterns, the volume fractions of austenite and ferrite in the steel annealed at 800 and 1000 °C were calculated as follows [23]:

(1) $$V_{\gamma }=\frac{\left(\frac{1}{n})\sum {}_{j=1}^{n}(I_{\gamma }^{j}/R_{\gamma }^{j}\right)}{\left(\frac{1}{n})\sum {}_{j=1}^{n}(I_{\gamma }^{j}/R_{\gamma }^{j})+(\frac{1}{n})\sum {}_{j=1}^{n}(I_{\alpha }^{j}/R_{\alpha }^{j}\right)}$$

where *V_γ_* is the volume fraction of austenite, *n* is the number of corresponding diffraction peaks used for calculation, *I* is the diffraction intensity and *R* is the scattering factor of the material. *R* can be calculated as follows:

(2) $$R=\frac{1}{V^{2}}\left[{\left|F\right|}^{2}P(\frac{1+\cos^{2}2\theta }{\sin\theta \sin2\theta })\right]e^{-2M}$$

where *V* is the volume of a single cell, *F* is the structure factor, *P* is the multiplicity factor and *e^−2M^* is the temperature factor. All parameters are given in [23]. The diffraction peaks used for calculation are indicated by arrows in Figure 2b. The calculated XRD results showed that the volume fraction of austenite reached 79.5% and 54.2% in the steel annealed at 1000 and 800 °C, respectively. The volume fraction of austenite increased with the annealing temperature. This result is consistent with that of the Thermo-Calc thermal simulation.

### 3.2. Tensile Property

**Figure 3:**
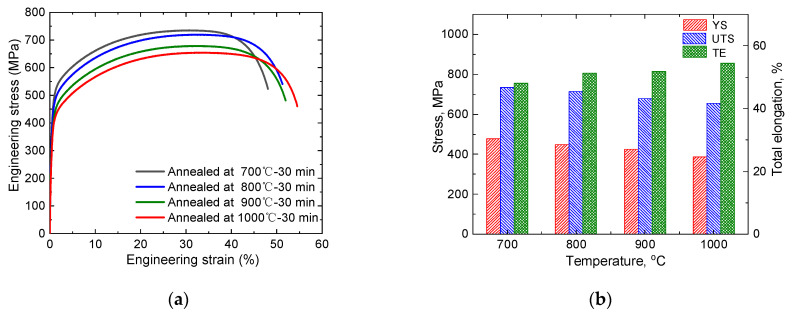
(**a**) Engineering stress–strain curves and (**b**) mechanical properties of the cold-rolled steel annealed at 700–1000 °C for 30 min.

Figure 3a shows the tensile properties of the experimental steel after cold rolling and annealing at different temperatures. All tensile curves showed excellent tensile properties with YS of 388–477 MPa, UTS over 650 MPa and TE as large as 47–55%. The detailed tensile properties are summarized in Figure 3b. With increasing annealing temperature, the YS and UTS of the steel decreased significantly. This phenomenon was primarily attributed to the increased austenite grain size during annealing at elevated temperatures. The TE increased with the annealing temperature, and the maximum elongation reached ~55%. When the steel was annealed at 800 °C, the product of strength and elongation was as high as ~37 GPa %. The superior mechanical properties of the steel met the requirements for third-generation automotive steel, and the density of the steel decreased due to the addition of the light Al element. The experimental steel was more competitive than other ordinary third-generation automotive steel.

### 3.3. Microstructure Evolution

**Figure 4:**
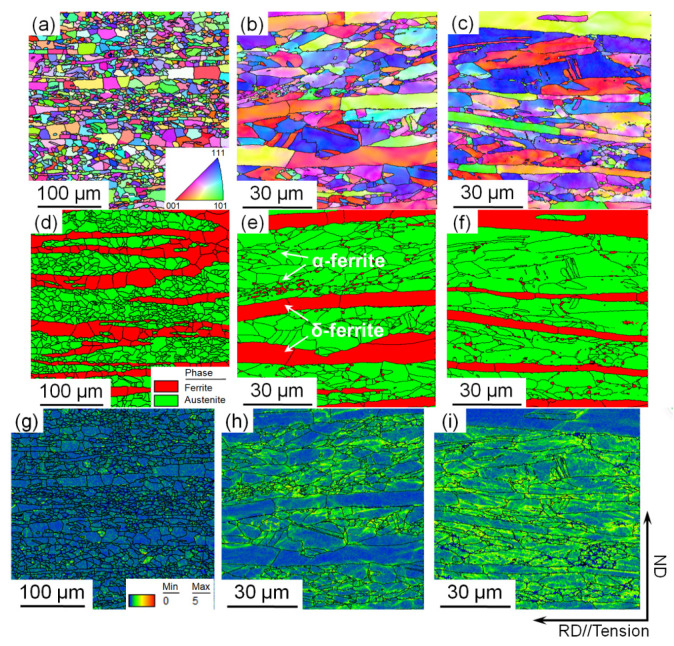
EBSD (**a**–**c**) inverse pole figures, (**b**–**f**) phase map and (**g**–**i**) kernel average misorientation of the samples annealed at 800 °C and stretched to strains of (**a**,**d**,**g**) 0, (**b**,**e**,**h**) 0.15 and (**c**,**f**,**i**) 0.3.

The microstructural evolution of the steel annealed at 800 °C was examined by EBSD. Figure 4a–c shows the inverse pole figures (IPFs) of the samples stretched to strains of 0, 0.15 and 0.3, respectively. Figure 4a indicates that the recrystallization of the sample was complete, the grain orientation was randomly distributed and no texture was generated. The austenite and ferrite were elongated with increasing strain, slight deformation texture could be observed and no deformation twins were observed in the austenite phase (Figure 4b,c). Figure 4d–f shows the corresponding phase map within the range of Figure 4a–c. These figures show that the steel annealed at 800 °C contained two phases of austenite and ferrite. Ferrite occurs in two types, coarse band-like δ-ferrite and fine granular α-ferrite. Fine α-ferrite disperses in the austenite grain boundaries, reported to precipitate from austenite during annealing [20]. However, ε-martensite was not observed, indicating that the transformation-induced plasticity (TRIP) mechanism did not occur in the austenite phase during the tensile deformation. Figure 4g–i shows the corresponding kernel average misorientation (KAM) map within the range of Figure 4a–c. The KAM value serves as an important index indicating local orientation gradients. The high KAM value of the deformed microstructures implied a wide distribution of misorientation, a high dislocation density and a greater accumulation of plastic deformation [24]. Figure 4h shows that the plastic deformation was mainly concentrated in the fine austenite and grain boundaries of the coarse δ-ferrite at the strain of 0.15. By contrast, the plastic deformation in the interior of coarse δ-ferrite was relatively small. Further increasing the strain to 0.3 gradually increased the deformation in the austenite. The interior of the coarse δ-ferrite also deformed significantly (Figure 4i).

### 3.4. Fracture Morphology

**Figure 5:**
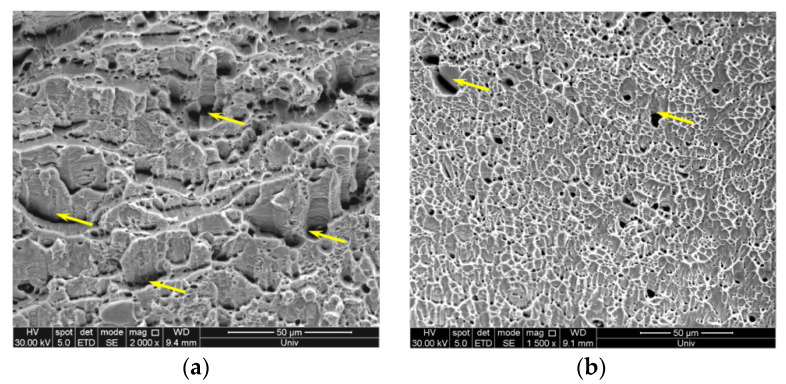
Scanning electron micrograph of the fracture morphology of the steel annealed at (**a**) 800 °C and (**b**) 1000 °C. Yellow arrows indicate the quasi-cleavage fracture morphology.

Figure 5a or Figure 5b show the fracture morphology of the steel annealed at 800 and 1000 °C, respectively. Two fracture morphologies, namely brittle quasi-cleavage fracture (yellow arrows) and ductile dimple fracture, were observed in both samples. The fracture morphology of the steel annealed at 800 °C contained more brittle quasi-cleavage fracture than that of the steel annealed at 1000 °C. Banded δ-ferrite was the main reason for quasi-cleavage fracture. Microcracks easily formed along the δ/γ boundaries during deformation and gradually expanded into the macroscopic fracture due to the plasticity mismatch between the band-like δ-ferrite and austenite [18]. The ductile dimple fracture was dominant in Figure 5b, and a small amount of quasi-cleavage fracture existed. With increasing annealing temperature, more austenite phase was formed in the annealed sample. In addition, the dimple fracture area of the sample increased, leading to increased plasticity of the steel.

## 4. Discussion

For steels containing austenitic phase, such as bainitic steel, Hadfield steel, TWIP steel and austenitic stainless steel, the SFE value of the austenite determines the deformation mechanism. The basic principle of SFE (*γ_SFE_*) calculation is based on the Gibbs free energy required to form martensite between two atomic layers [25]. Based on a modified Olson–Cohen thermodynamical model that is widely used to calculate SFE, the SFE is expressed as follows [26,27]:

(3) $$\gamma_{SFE}=2\rho \Delta G^{\gamma \to \varepsilon }+2\sigma$$

where *ρ* is the molar surface density along the {1 1 1} planes, Δ*G_γ→ε_* is the molar Gibbs energy of the austenite to ε-martensite phase transformation and *σ* is the interfacial energy per unit area of the phase boundary. The parameters used to calculate the SFE are provided in [26]. The SFE of the steel calculated using the nominal composition was 28.6 mJ/m^2^, which was within the range in which the TWIP effect can occur [28]. However, according to the results of EBSD, neither the TWIP nor the TRIP effect happened during the tensile deformation. We infer that the segregation of the alloying elements in the ferrite and austenite phase changed the alloying composition of austenite, ultimately affecting the deformation mechanism.

**Figure 6:**
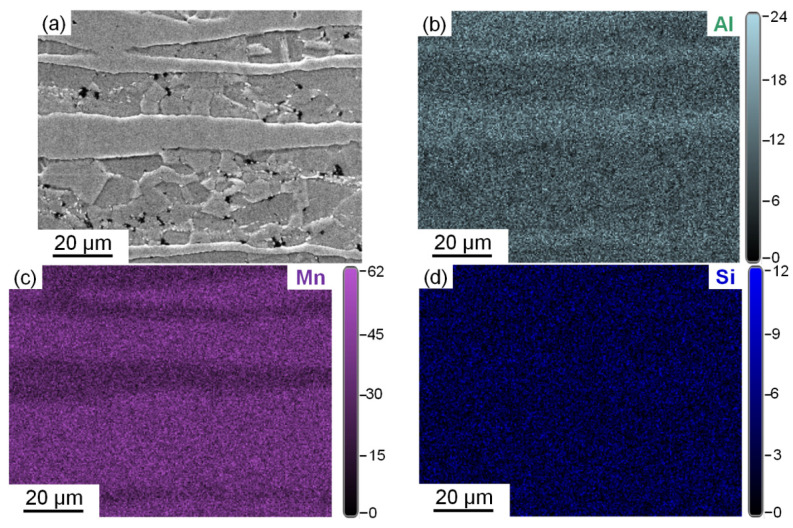
(**a**) Scanning electron micrograph of the microstructure morphology of the sample annealed at 800 °C and EDS mapping images of the distribution of (**b**) Al, (**c**) Mn and (**d**) Si.

Figure 6 shows the results of the EDS mapping analysis of the steel after annealing at 800 °C. Figure 6a shows the scanning electron micrograph of the morphology within the range of surface scanning, in which the band-like structure was δ-ferrite, and the rest was austenite. Figure 6b–d shows the distribution of Al, Mn and Si in the scanning range. Al was segregated in the ferrite phase, and Mn was segregated in the austenite phase. By contrast, Si showed no obvious segregation. Al and Mn alloying elements were the key elements affecting the SFE, and their segregation would inevitably affect the SFE of the austenite phase.

**Figure 7:**
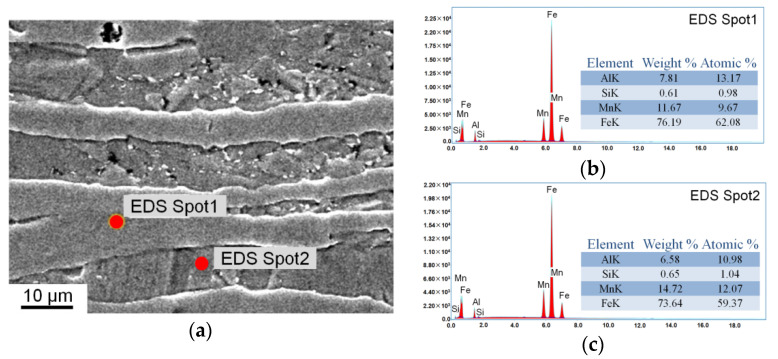
(**a**) Scanning electron micrograph of the location of the EDS spots and elemental analysis result of (**b**) spot 1 and (**c**) spot 2.

The alloying composition of austenite in the steel was measured by EDS point scanning (Figure 7). Figure 7a shows the detected points during the EDS analysis, where spot 1 was located in the δ-ferrite phase and spot 2 was located in the austenite phase. Elemental analysis results of spots 1 and 2 are shown in Figure 7b or Figure 7c, respectively. The chemical compositions measured by EDS had slight errors in detection at different points. Therefore, the chemical composition of each phase was measured thrice, and the average alloying compositions of δ-ferrite were as follows: Mn, 11.7 wt%; Al, 7.8 wt%; and Si, 0.6 wt%. For the austenite, Mn was 14.7 wt%, Al was 6.6 wt% and Si was 0.65 wt%. Element C is another major element that significantly affected the SFE. However, the content of C in the austenite could not be determined by EDS. The XRD results showed that the steel only contained ferrite and austenite phases after annealing at 800 °C, and the volume fraction of austenite in the steel was 54.2%. The C content in the ferrite phase was extremely low at room temperature, and this value could be ignored. We infer that all C atoms in the steel were dissolved in the austenite. The calculated C content in the austenite was 0.37 wt%. After considering element segregation and two-phase proportion in the steel, the calculated SFE was 39.7 mJ/m^2^. This value was significantly higher than the value calculated by nominal composition (28.6 mJ/m^2^). 

The formation of deformation twinning is the competition between the critical twinning stress (*σ_tw_*) and flow stress of the steel. When the flow stress was higher than the *σ_tw_*, the TWIP effect occurred. When the *σ_tw_* was greater than the maximum flow stress, the TWIP effect could not occur in the steel. The *σ_tw_* was related to SFE and grain size and calculated as follows [29]:

(4) $$\sigma_{tw}=(1/m)[(\gamma /b)+(K_{tw}^{H-P}/\sqrt{D})]$$

where *m* is the Schmidt factor (0.326 [30]), *γ* is the SFE, *b* is the Burgers vector (2.5 × 10^−10^ m), $K_{\mathrm{tw}}^{H-P}$ is the twin formation coefficient (356 MPa m^1/2^ [31]) and D is the grain size. According to the statistical result of EBSD, the average grain size of the austenite phase was 6.6 μm when the steel was annealed at 800 °C. The calculated *σ_tw_* of the steel was 897 MPa. However, the maximum flow stress of the steel was only 719 MPa, which failed to trigger the formation of deformation twins. Therefore, deformation twins were not observed in the deformation microstructure of the steel. Dislocation slip was the primary deformation mechanism of the steel.

## 5. Conclusions

The tensile deformation behavior of the Fe–12Mn–7Al–0.2C–0.6Si steel after cold rolling and annealing was investigated at room temperature. The following conclusions were drawn:

(1) Equiaxed austenite and band-like δ-ferrite phases were found in the microstructure of low-density Fe–12Mn–7Al–0.2C–0.6Si steel after cold rolling and annealing. The volume fraction of austenite increased with the annealing temperature.

(2) With increasing annealing temperature, the grain size of austenite increased, resulting in a decrease in the yield and tensile strength. However, plasticity increased due to the increased austenite content.

(3) At initial strains, the deformation is mainly concentrated at the fine austenite and grain boundaries of the coarse δ-ferrite, and the interior of coarse δ-ferrite gradually deformed with further increase in the strain to 0.3. No TWIP or TRIP effect occurred during the tensile deformation.

(4) Al was segregated in the δ-ferrite, and Mn was segregated in the austenite. Considering the element segregation and two-phase proportion fraction, the austenite SFE of the steel after annealing at 800 °C was 39.7 mJ/m^2^. The critical stress required to produce deformation twins was significantly higher than the maximum flow stress of the steel.

## Data Availability

The study did not report any data.
